# Plasmid profiling and incompatibility grouping of multidrug resistant *Salmonella enterica* serovar Typhi isolates in Nairobi, Kenya

**DOI:** 10.1186/s13104-019-4468-9

**Published:** 2019-07-16

**Authors:** Winnie C. Mutai, Peter G. Waiyaki, Samuel Kariuki, Anne W. T. Muigai

**Affiliations:** 1 Department of Medical Microbiology, School of Medicine, University of Nairobi, Nairobi, Kenya; 2 Centre for Microbiology Research, Kenya Medical Research Institute, Nairobi, Kenya; 3 School of Biological Sciences, Jomo Kenyatta University of Agriculture and Technology, Juja, Kenya

**Keywords:** Multidrug resistant *S*. Typhi, Plasmid incompatibility grouping, Conjugation

## Abstract

**Objectives:**

Plasmids harbour antibiotic resistance genes which contribute to the emergence of multidrug resistant pathogens. We detected the presence of plasmids in multidrug resistant *Salmonella enterica* serovar Typhi (*S*. Typhi) isolates from our previous study and consequently determined their incompatibility groups and possibility of conjugation transmission. Plasmids were extracted from 98 multidrug resistant *S*. Typhi isolates based on alkaline lysis technique. Plasmid incompatibility grouping was established by PCR replicon typing using 18 pairs of primers to amplify FIA, FIB, FIC, HI1, HI2, I1-Iγ, L/M, N, P, W, T, A/C, K, B/O, X, Y, F and FIIA replicons. Antibiotic resistance phenotypes were conjugally transferred from *S*. Typhi isolates with plasmids to *Escherichia coli* K12F strain devoid of plasmids.

**Results:**

Approximately 79.6% of the MDR *S*. Typhi isolates were related to the existence of plasmids. We detected 93.6% of plasmids belonging to incompatibility (Inc) group HI1. The other incompatibility groups identified included IncFIC (16.7%), IncP (1.3%), and IncI1 (1.3%) which appeared together with Inc HI1. MDR *S*. Typhi isolated carried a homologous plasmid of incompatibility group HI1 most of which transferred the resistance phenotypes of ampicillin, tetracycline and chloramphenicol to the transconjugants.

**Electronic supplementary material:**

The online version of this article (10.1186/s13104-019-4468-9) contains supplementary material, which is available to authorized users.

## Introduction

Plasmids have been defined as circular double-stranded deoxyribonucleic acid (DNA) molecules that exist and replicate independently within a bacterial cell or can be integrated into the bacterial chromosome. Plasmids serve as vectors for lateral mobility of genetic information between bacterial cell consequently promoting their spread and sustainability within a bacterial niche under changing environmental stimuli [1]. Genes encoding different traits in a bacteria are lugged in plasmids and are expressed when the bacteria encounters a contentious environment especially those inflicted by human activities including antibiotic use [2]. This phenomenon has played a big role in evolution of antibiotic resistance bacteria especially among gram-negative Enterobacteriaceae contributing to treatment failure and persistence of infectious diseases in the population [3, 4].

Plasmids classification based on their stability during transmission is evaluated by exploring their incompatibility categories. Incompatibility grouping represents “the inability of two plasmids to coexist stably over a number of generations in the same bacterial cell line” such that plasmids incompatible to each other are assigned the same group [5]. This mechanism was first described in the early 1970s by Datta and Hedges and about 30 incompatibility groups of plasmids have so far been recognized in the family of Enterobacteriaceae [6]. Further, based on genetic relatedness the incompatibility groups have been sub-grouped into four major clusters: IncF group (IncF, IncS, IncC, IncD, IncJ); IncP group (IncP, IncU, IncM, IncW); Ti group (IncX, IncH, IncN, IncT) and IncI group (IncI, IncB and IncK) [7]. Most of these plasmids especially those of incompatibility groups IncA/C, B/O, L/M, HI1, HI2, I1, N, F, and P are associated with multidrug resistant (MDR) isolates from clinical, animal and environmental samples [4, 8–10]. MDR *S.* Typhi mostly, habors plasmid of IncHI, however other incompatibility groups including IncF, IncP, and IncB/O plasmids have also been identified [11, 12].

With the rapidly evolving molecular techniques, more advanced plasmid analysis tools with a high discriminatory power and a quick turnaround time are constantly being developed. These tools provide more precise results for epidemiological investigations. However in settings were these tools are not accessible simple molecular techniques including plasmid replicon typing still provides an insight on the role of plasmids in transmitting antibiotic resistance. Therefore in this work we detected the existence of plasmids in previously isolated MDR *S*. Typhi strains [13] and analysed the plasmids by means of incompatibility grouping adopting a PCR based replicon typing protocol illustrated by Carattoli et al. [14]. We further screened the plasmids for their ability to transfer antibiotic resistance to an *E. coli* strain short of plasmids.

## Main text

### Methods

#### Plasmid extraction

The 98 archived MDR *S.* Typhi isolates from our previous study [13] where sub-cultured onto MacConkey agar. Discrete colonies were then transferred into 3 ml of Luria broth and incubated at 37 °C overnight on a shaker (250 rpm). After 18 h incubation, liquid culture was transferred to a 1.5 ml eppendorf tube and centrifuged to harvest the cells at the bottom of the tube. Plasmids were extracted from the sediment cell pellet using alkaline lysis protocol described by Birnboim and doly [15]. *E. coli* strain 39R861 (NCTC 50192) with known plasmids of molecular size of 98, 42, 23.9 and 4.6 MDa size markers was used as the control strain. The products were analysed on 1.5% agarose gel.

#### PCR plasmid incompatibility grouping

Plasmid incompatibility grouping was based on the method previously described by Carattoli et al. [14]. Briefly, 18 pairs of primers targeting replicons FIA, FIB, FIC, HI1, HI2, I1, L/M, N, P, W, T, A/C, K, B/O, X, Y, F and FIIA were used for conventional amplification in a five multiplex- and three simplex-PCR regimen. The amplicons were visualized under UV transilluminator in a electrophoresis stained with ethidium bromide. The incompatibility groups were identified based on the amplicon sizes described by Carattoli et al. [14].

#### Transferable resistance plasmids

In-vitro conjugation experiment on transferable plasmids were performed according to the method described by Walia et al. [16], *E. coli* K12F devoid of plasmids and resistant to nalidixic acid was used as the recipient organism for this experiment. From the MDR *S.* Typhi isolates we selected 68 isolates resistant to ampicillin and sensitive to nalidixic acid. The transconjugants were then screened on MacConkey agar plates integrated with nalidixic acid (32 mg/l) and ampicillin (32 mg/l). To determine the transferable resistance-encoding plasmids, plasmid DNA were extracted from the transconjugants and visualised on a 1% agarose gel electrophoresis.

### Results

#### Plasmid analysis

**Fig. 1:**
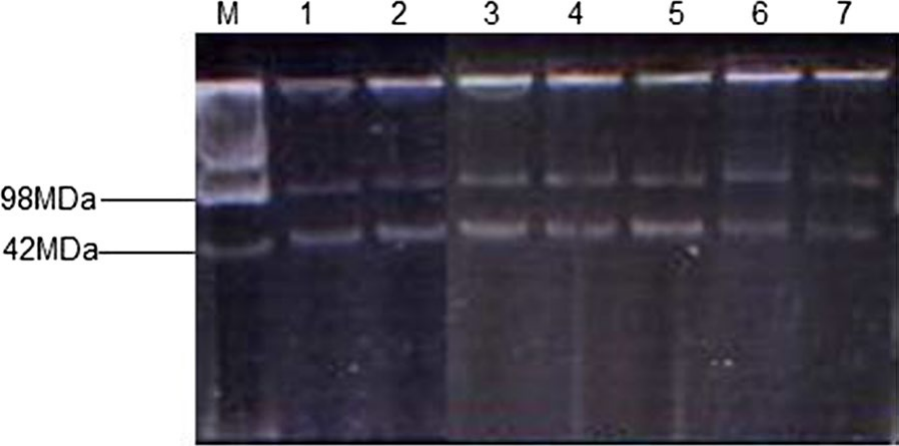
Plasmid profile from selected multi-drug resistance isolates. Lane M—plasmids of the reference strain *E. coli* R39 (NCTC 50192); Lanes 1–7 plasmids of MDR *S*. Typhi isolates

**Fig. 2:**
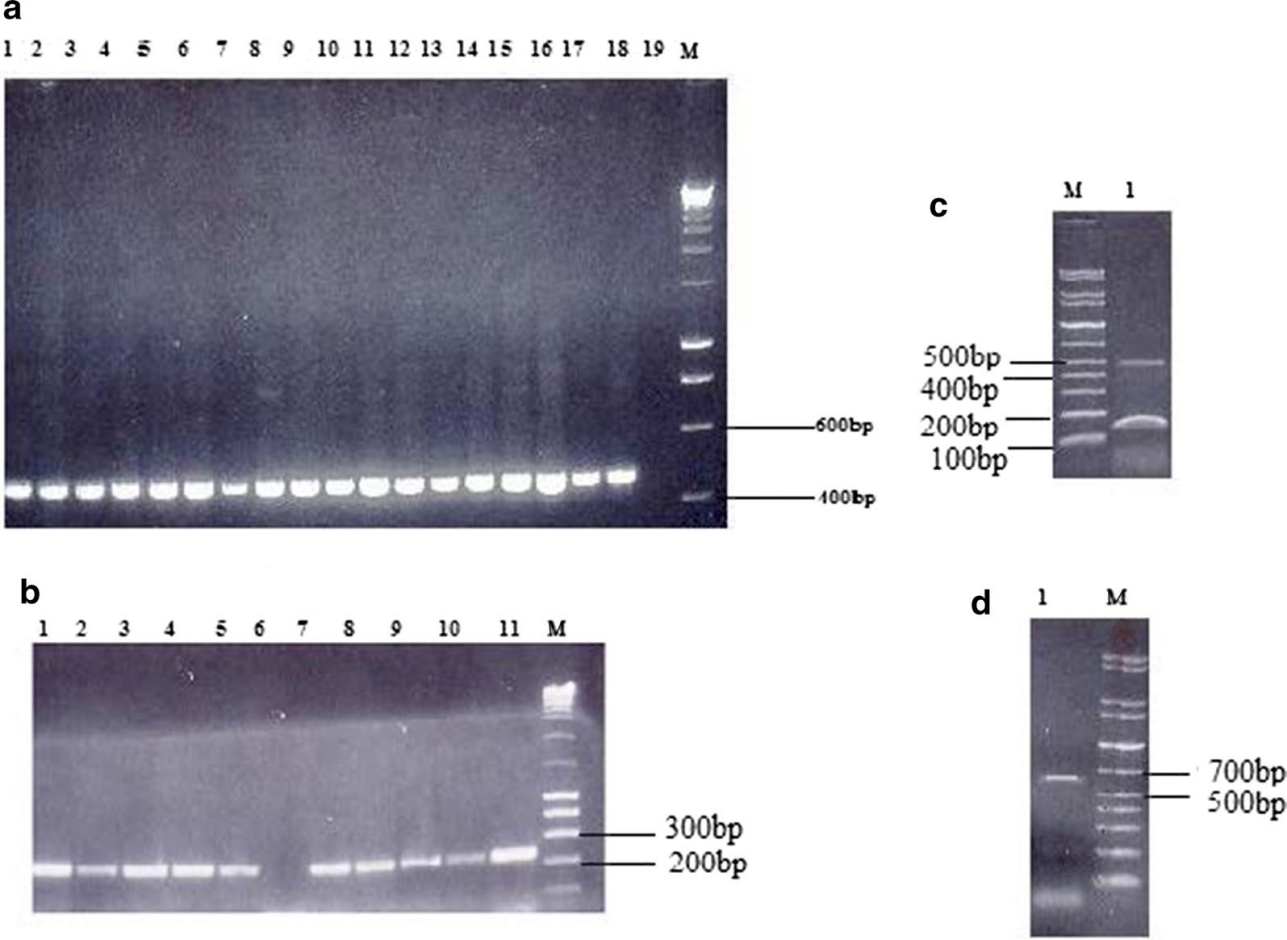
Gel electrophoresis showing the amplicon products of Incompatibility groups IncHI1, IncFIC, IncI1 and IncP. **a** Lanes 1–18—471-bp IncHI1 amplicon, lane 19—*S*. Typhi sensitive to all the antibiotics tested, lane M-200 bp MW ladder; **b** Lane 1, 2, 3, 4, 5, 7, 8, 9, 10, 11—IncFIC (262 bp), lane 6 MDR *S*. Typhi without IncFIC; **c** Lane 1—IncI1 (139 bp); **d** Lane 1—IncP (534 bp) lane M 100 bp marker

Approximately 79.6%% of the 98 MDR *S.* Typhi isolates expressed a larger 98 MDa plasmid and 54% a smaller 42 MDa plasmid as shown in Table 1 and Fig. 1 (lanes 1–7). IncHI1 plasmid predominated among the replicon types identified (73/78, 93.6%) as represented in Table 1 and Fig. 2a, the other replicon types observed included IncFIC (13/78, 16.7%) (Fig. 2b), IncI1 (1/78, 1.3%) (Fig. 2c) and IncP (1/78, 1.3%) (Fig. 2d). Three sets of combined replicon types were observed IncHI1 + IncFIC (11/78, 14.1%), IncHI1 + IncFIC + IncP (1/78, 1.3%) and IncHI1 + IncFIC + IncI (1/78, 1.3%) as shown in Table 1. Plasmids and the replicon types prevailed among the isolates that exhibited resistance to tetracycline, chloramphenicol and ampicillin. Interestingly, we also observed the reduction of IncHI1 replicon types during the study period (Table 1).

**Table 1:** Summary of the distribution of plasmid sizes, incompatibility groups and transconjugants in relation to the resistance phenotypes identified

Resistance phenotype	Plasmid size 42 MDa N (%)	Plasmid size 98 MDa N (%)	Incompatibility groups (N)			Resistance pattern of transconjugants (N)
			2004	2005	2006	
AMC,TET,C,AMP (N = 10)	1 (1.0%)	4 (4.1%)	HI(4)	–	–	AMC,TET,C,AMP(5) TET,C,AMP(1) AMC,AMP(1) TET(1) NT(2)
CF,TET,C,AMP (N = 2)	0 (0.0%)	2 (2.0%)	HI(1)	HI(1)	–	TET,C,AMP(1) TET,AMP (1) NT(1)
TET,C,AMP (N = 71)	52 (53.0%)	70 (71.4%)	HI(25), FIC(6), I(1)	HI(24), FIC(4), P(1)	HI(17), FIC(3)	TET,C,AMP(23) TET,AMP(4) C,AMP(1) TET(4) C(2) AMP(1) NT(21)
Others (N = 11)	0 (0.0%)	2 (2.0%)	HI(1)	–	–	–

*AMP* ampicillin, *AMC* amoxycillin–clavulanic acid, *CF* ciprofloxacin, *C* chloramphenicol, *TET* tetracycline, *NT* not transferable

#### Conjugation experiments

Transconjugants grew on plates containing both ampicillin (32 mg/l) and nalidixic acid (32 mg/l). Of the 68 MDR *S.* Typhi isolates selected for conjugation, 45 (66.2%) isolates transferred resistance of one or more antimicrobials to recipient *E. coli* K12 (Table 1). Twenty-eight (41.2%) isolated transferred full resistance phenotype with majority transfer being that of tetracycline, chloramphenicol and ampicillin (23/28, 82.1%). Additionally 23 (33.8%) isolates did not transfer any of the resistant traits to the recipient. The acquisition of the antibiotic resistance phenotypes in the transconjugants was related to the presence of 98 MDa size plasmids and in some isolates a smaller plasmid of 42 MDa (Additional file 1: Fig. S1; Additional file 2: Fig. S2).

### Discussion

In this current study 79.6% of the MDR *S*. Typhi carried large self-transmissible plasmids of > 98 MDa. Additionally the main replicon identified among the plasmids analysed were those of incompatibility group HI1. Plasmid of this incompatibility group could be accounting for the persistence and spread of MDR *S*. Typhi isolates. Since the first report of an IncHI1 plasmid encoding MDR *S*. Typhi isolates in Mexico City in 1972, many studies globally are currently reporting similar isolates among MDR *S.* Typhi and other Enterobacteriaceae [17–22].

The most common resistance phenotypes among the isolates in this study were linked to tetracycline, ampicillin and chloramphenicol resistance and were significantly associated with the presence of plasmids mainly of IncHI1. Plasmids of incompatibility group HI1 (IncHI1) have been shown to encode multiple antibiotic resistances specifically to ampicillin, chloramphenicol, trimethoprim, sulfonamides, streptomycin and tetracyclines and most of which are associated with outbreaks [17, 23, 24]. The results from the current study show that it is likely that the same plasmid of IncHI1 could be circulating and spreading widely within the MDR strains of *S*. Typhi however, this would have been well supported by comparative analysis to detect the clustering of these plasmids.

These homologous plasmids among the isolates analysed may have been acquired from other *Salmonella enterica* strains or from other Enterobacteriaceae as has been previously investigated that IncHI1 plasmid can be transferred between the Enterobacteriaceae or naturally through horizontal gene transfer among *S*. Typhi strains [25–27]. More studies based on sequence analysis of the conjugative plasmids reveal that these plasmids are closely related and share related resistance genes suggesting that this plasmids that confer antibiotic resistance may have been transferred between these serovars [28, 29]. It is unfortunate that this study did not go further to perform plasmid sequence to prove this hypothesis.

Other than IncHI1 we further identified other replicons including IncI1, IncP, and IncFIC all of which co-existed together with Inc HI1. Plasmids belonging to the IncI1 family are considered as virulence plasmids encoding type IV pili that play a role in attachment of the bacteria to the host cells. However in this context IncI1 plasmids have been described in *E. coli*, *Salmonella* and other Enterobacteriaceae as vectors of resistant genes that code for extended-spectrum β-lactamase (ESBL) predominantly *bla*_CTX-M_, *bla*_TEM-1_ and *bla*_SHV_ and to a lesser extent plasmid mediated AmpC β-lactamase gene (*bla*CMY-2) that code for cephamycin and carbapenem resistance [30–32]. IncP plasmids on the other hand are widely distributed in the environment habitat including water, soil and waste water treatment plants among Enterobacteriaceae family and carry genes that confer antibiotics and heavy metal resistance [33–37]. Recently, *Salmonella enterica* serovar Typhimurium strain resistant to colistin and carried by an IncP plasmid was isolated from a healthy individual. Additionally conjugation experiments from this same study indicated that the plasmids played a role in dissemination of *mcr*-*1* gene responsible for colistin resistance indicating that the individual may have been a carrier facilitating the spread of resistant strains [38].

Conjugation experiment indicated that 66.2% of the MDR selected for conjugation transferred some or all of the resistance phenotypes to *E. coli* K12. It is certain that the conjugal transfer of antibiotic resistance to the transconjugants may have occurred via plasmids consequently impelling the spread of antibiotic resistance.

Antibiotic resistance in MDR *S*. Typhi mainly to first line antibiotics is encoded by plasmids. In this study we show that conjugative plasmids especially those of Inc HI1 were relevant in disseminating antibiotic resistance predominantly the first line antibiotics. Knowing the predominant replicon type forms a basis for future studies with approaches intended to construct genes that block horizontal transfer of this group of plasmids which have been successfully utilized to counter other incompatibility group of plasmids [39, 40].

## Limitations

The information gathered in this study affirms the role of plasmids in driving antibiotic resistance however comparative genomic analysis addressing the evolution of these plasmids categorically how the loss, gain and persistence of certain groups of plasmids facilitates the dissemination of antibiotic resistance bacterial pathogens would have supported this study.

## Additional files

**Additional file 1: Fig. S1.** Plasmids extracted from the transconjugants. Lane M plasmids of *E. coli* 39 (NCTC 50192); Lane 1–10 transconjugants resistant to chloramphenicol, tetracycline and ampicillin; Lane 5 non-conjugative transconjugant.

**Additional file 2: Fig. S2.** MacConkey culture plates showing the morphology of the recipient, donor and the transconugant. (A): Colonies of the recipient strain (*E. coli* K12) in presence of nalidixic acid; (B): Colonies of the donor strain (*S.* Typhi) on culture plate containing ampicillin; (C): Colonies of transconjugants on culture plate containing both ampicillin and nalidixic acid.

## Data Availability

The datasets analysed in this study are provided as supporting documents.

## Abbreviations

MDR: multidrug resistant
Inc: incompatibility group
NCTC: National Collection of Type Cultures
ESBL: extended-spectrum β-lactamase resistance
MDa: megadalton

## References

[1] Baquero F, Tedim AP, Coque TM (2013). Antibiotic resistance shaping multi-level population biology of bacteria. Front Microbiol.

[2] Bennett PM (2008). Plasmid encoded antibiotic resistance: acquisition and transfer of antibiotic resistance genes in bacteria. Br J Pharmacol.

[3] Wang J, Stephan R, Zurfluh K, Hächler H, Fanning S (2014). Characterization of the genetic environment of bla ESBL genes, integrons and toxin–antitoxin systems identified on large transferrable plasmids in multi-drug resistant *Escherichia coli*. Front Microbiol.

[4] Chen W, Fang T, Zhou X, Zhang D, Shi X, Shi C (2016). IncHI2 plasmids are predominant in antibiotic-resistant *Salmonella* isolates. Front Microbiol.

[5] Thomas Christopher M. (2014). Plasmid Incompatibility. Molecular Life Sciences.

[6] Datta N, Hedges RW (1973). R factors of compatibility group A. J Gen Microbiol.

[7] Waters VL (1999). Conjugative transfer in the dissemination of beta-lactam and aminoglycoside resistance. Front Biosci.

[8] Glenn LM, Lindsey RL, Folster JP, Pecic G, Boerlin P, Gilmour MW (2013). Antimicrobial resistance genes in multidrug-resistant *Salmonella enterica* isolated from animals, retail meats, and humans in the United States and Canada. Microb Drug Resist.

[9] Poole TL, Edrington TS, Brichta-Harhay DM, Carattoli A, Anderson RC, Nisbet DJ (2009). Conjugative transferability of the A/C plasmids from *Salmonella enterica* isolates that possess or lack *bla*_CMY_ in the A/C plasmid backbone. Foodborne Pathog Dis.

[10] Lyimo B, Buza J, Subbiah M, Temba S, Kipasika H, Smith W (2016). IncF plasmids are commonly carried by antibiotic resistant *Escherichia coli* isolated from drinking water sources in northern Tanzania. Int J Microbiol.

[11] Phan M-D, Kidgell C, Nair S, Holt KE, Turner AK, Hinds J (2009). Variation in *Salmonella enterica* serovar typhi IncHI1 plasmids during the global spread of resistant typhoid fever. Antimicrob Agents Chemother.

[12] Mirza S, Kariuki S, Mamun KZ, Beeching NJ, Hart CA (2000). Analysis of plasmid and chromosomal DNA of multidrug-resistant *Salmonella enterica* serovar typhi from Asia. J Clin Microbiol.

[13] Mutai WC, Muigai AWT, Waiyaki P, Kariuki S (2018). Multi-drug resistant *Salmonella enterica* serovar Typhi isolates with reduced susceptibility to ciprofloxacin in Kenya. BMC Microbiol.

[14] Carattoli A, Bertini A, Villa L, Falbo V, Hopkins KL, Threlfall EJ (2005). Identification of plasmids by PCR-based replicon typing. J Microbiol Methods.

[15] Birnboim HC, Doly J (1979). A rapid alkaline extraction procedure for screening recombinant plasmid DNA. Nucleic Acids Res.

[16] Walia SK, Madhavan T, Chugh TD, Sharma KB (1987). Characterization of self-transmissible plasmids determining lactose fermentation and multiple antibiotic resistance in clinical strains of *Klebsiella pneumoniae*. Plasmid.

[17] Kariuki S, Revathi G, Muyodi J, Mwituria J, Munyalo A, Mirza S (2004). Characterization of multidrug-resistant typhoid outbreaks in Kenya. J Clin Microbiol.

[18] Wain J, Diem Nga LT, Kidgell C, James K, Fortune S, Song Diep T (2003). Molecular analysis of incHI1 antimicrobial resistance plasmids from *Salmonella* serovar Typhi strains associated with typhoid fever. Antimicrob Agents Chemother.

[19] Fica A, Fernandez-beros ME, Aron-hott L, Rivas A, Dottone K, Chumpitaz J (1997). Antibiotic-resistant *Salmonella typhi* from two outbreaks: few ribotypes and IS *200* Types Harbor Inc HI1 Plasmids. Microb Drug Resist.

[20] Harnett N, McLeod S, AuYong Y, Wan J, Alexander S, Khakhria R (1998). Molecular characterization of multiresistant strains of *Salmonella typhi* from South Asia isolated in Ontario, Canada. Can J Microbiol.

[21] Buckle GC, Walker CLF, Black RE, Lee K, Crump J, Luby S (2012). Typhoid fever and paratyphoid fever: systematic review to estimate global morbidity and mortality for 2010. J Glob Health.

[22] Shanahan PM, Jesudason MV, Thomson CJ, Amyes SG (1998). Molecular analysis of and identification of antibiotic resistance genes in clinical isolates of *Salmonella typhi* from India. J Clin Microbiol.

[23] Breiman RF, Cosmas L, Njuguna H, Audi A, Olack B, Ochieng JB (2012). Population-based incidence of typhoid fever in an urban informal settlement and a rural area in Kenya: implications for typhoid vaccine use in Africa. PLoS ONE.

[24] Yan M, Li X, Liao Q, Li F, Zhang J, Kan B (2016). The emergence and outbreak of multidrug-resistant typhoid fever in China. Emerg Microbes Infect.

[25] Holt KE, Phan MD, Baker S, Duy PT, Nga TVT, Nair S (2011). Emergence of a globally dominant IncHI1 plasmid type associated with multiple drug resistant typhoid. PLoS Negl Trop Dis.

[26] Kubasova T, Cejkova D, Matiasovicova J, Sekelova Z, Polansky O, Medvecky M (2016). Antibiotic resistance, core-genome and protein expression in IncHI1 plasmids in *Salmonella* Typhimurium. Genome Biol Evol.

[27] Hradecka H, Karasova D, Rychlik I (2008). Characterization of *Salmonella enterica* serovar Typhimurium conjugative plasmids transferring resistance to antibiotics and their interaction with the virulence plasmid. J Antimicrob Chemother.

[28] Holt KE, Thomson NR, Wain J, Phan MD, Nair S, Hasan R (2007). Multidrug-resistant *Salmonella enterica* serovar paratyphi A harbors IncHI1 plasmids similar to those found in serovar typhi. J Bacteriol.

[29] Phan M-D, Wain J (2008). IncHI plasmids, a dynamic link between resistance and pathogenicity. J Infect Dev Ctries.

[30] Tagg KA, Iredell JR, Partridge SR (2014). Complete sequencing of IncI1 sequence type 2 plasmid pJIE512b indicates mobilization of blaCMY-2 from an IncA/C plasmid. Antimicrob Agents Chemother.

[31] van Boxtel R, Wattel AA, Arenas J, Goessens WHF, Tommassen J (2017). Acquisition of carbapenem resistance by plasmid-encoded-AmpC-expressing *Escherichia coli*. Antimicrob Agents Chemother.

[32] Smith H, Bossers A, Harders F, Wu G, Woodford N, Schwarz S (2015). Characterization of epidemic IncI1-Iγ plasmids harboring ambler class A and C genes in Escherichia coli and *Salmonella enterica* from animals and humans. Antimicrob Agents Chemother.

[33] Zhao F, Feng Y, Lü X, McNally A, Zong Z (2017). IncP plasmid carrying colistin resistance gene mcr-1 in *Klebsiella pneumoniae* from Hospital Sewage. Antimicrob Agents Chemother.

[34] Sen D, Van der Auwera GA, Rogers LM, Thomas CM, Brown CJ, Top EM (2011). Broad-host-range plasmids from agricultural soils have IncP-1 backbones with diverse accessory genes. Appl Environ Microbiol.

[35] Schluter A, Heuer H, Szczepanowski R, Forney LJ, Thomas CM, Pühler A (2003). The 64 508 bp IncP-1 antibiotic multiresistance plasmid pB10 isolated from a waste-water treatment plant provides evidence for recombination between members of different branches of the IncP-1 group. Microbiology.

[36] Schlüter A, Szczepanowski R, Pühler A, Top EM (2007). Genomics of IncP-1 antibiotic resistance plasmids isolated from wastewater treatment plants provides evidence for a widely accessible drug resistance gene pool. FEMS Microbiol Rev.

[37] Heuer H, Smalla K (2012). Plasmids foster diversification and adaptation of bacterial populations in soil. FEMS Microbiol Rev.

[38] Lu X, Hu Y, Luo M, Zhou H, Wang X, Du Y (2017). MCR-1.6, a new MCR variant carried by an IncP plasmid in a colistin-resistant *Salmonella enterica* Serovar Typhimurium isolate from a healthy individual. Antimicrob Agents Chemother.

[39] Kamruzzaman M, Shoma S, Thomas CM, Partridge SR, Iredell JR (2017). Plasmid interference for curing antibiotic resistance plasmids in vivo. PLoS ONE.

[40] Getino M, Palencia-Gándara C, Garcillán-Barcia MP, de la Cruz F (2017). PifC and Osa, plasmid weapons against rival conjugative coupling proteins. Front Microbiol.

