# Supplementary material for: Plasmid profiling and incompatibility grouping of multidrug resistant Salmonella enterica serovar Typhi isolates in Nairobi, Kenya
Source: BMC Res Notes. 2019 Jul 16;12:422. doi: 10.1186/s13104-019-4468-9 (PMC6636098; doi:10.1186/s13104-019-4468-9)
Supplement: Supplementary file 1 — Additional file 1: Fig. S1. Plasmids extracted from the transconjugants. Lane M plasmids of E. coli 39 (NCTC 50192); Lane 1–10 transconjugants resistant to chloramphenicol, tetracycline and ampicillin; Lane 5 non-conjugative transconjugant. [file 13104_2019_4468_MOESM1_ESM.pdf]

Additional file 1: Fig S1

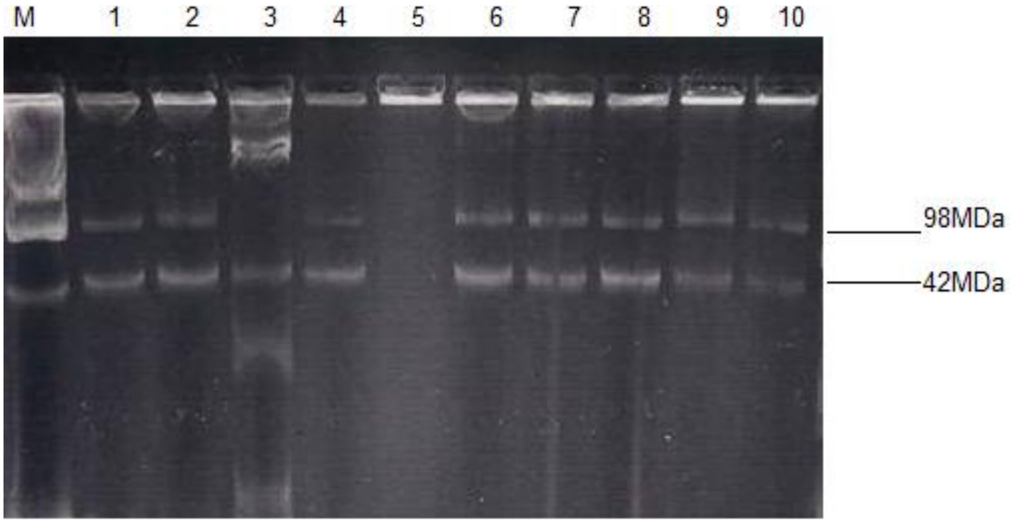

Plasmids extracted from the transconjugants. Lane M plasmids of *E. coli* 39 (NCTC 50192); Lane 1-10 transconjugants resistant to chloramphenicol, tetracycline and ampicillin; Lane 5 non-conjugative transconjugant.
