# Supplementary material for: Plasmid profiling and incompatibility grouping of multidrug resistant Salmonella enterica serovar Typhi isolates in Nairobi, Kenya
Source: BMC Res Notes. 2019 Jul 16;12:422. doi: 10.1186/s13104-019-4468-9 (PMC6636098; doi:10.1186/s13104-019-4468-9)
Supplement: Supplementary file 2 — Additional file 2: Fig. S2. MacConkey culture plates showing the morphology of the recipient, donor and the transconugant. (A): Colonies of the recipient strain (E. coli K12) in presence of nalidixic acid; (B): Colonies of the donor strain (S. Typhi) on culture plate containing ampicillin; (C): Colonies of transconjugants on culture plate containing both ampicillin and nalidixic acid. [file 13104_2019_4468_MOESM2_ESM.pdf]

Additional file 2: Fig S2

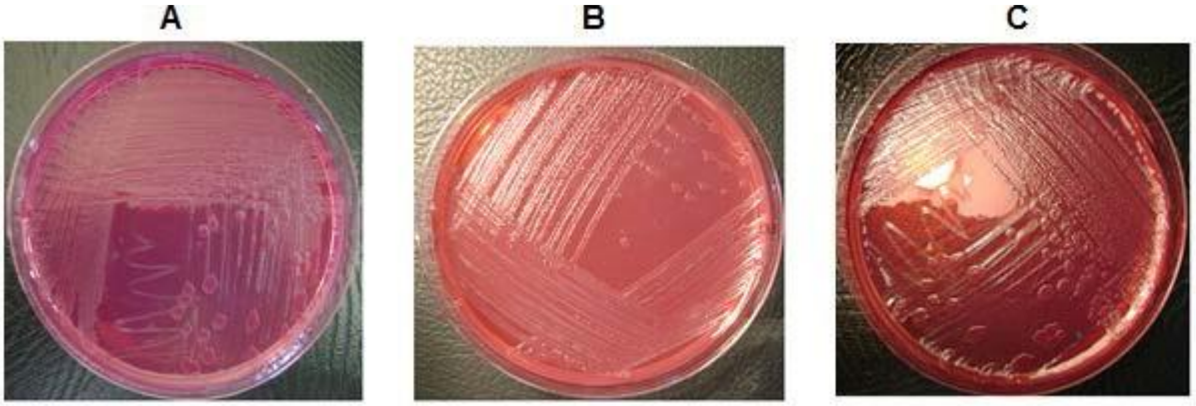

MacConkey culture plates showing the morphology of the recipient, donor and the transconugant. (A): Colonies of the recipient strain (*E. coli* K12) in presence of nalidixic acid; **(B)**: Colonies of the donor strain (*S.* Typhi) on culture plate containing ampicillin; **(C):** Colonies of transconjugants on culture plate containing both ampicillin and nalidixic acid.
